# Predictors and Utilization of Health Institution Services for Childbirth among Mothers in a Southern Nigerian City

**DOI:** 10.1155/2021/6618676

**Published:** 2021-12-07

**Authors:** Kazeem Arogundade, June Sampson, Elizabeth Boath, Ubong Akpan, Olaposi Olatoregun, Oluwayemisi Femi-Pius, Jude Orjih, Barinaadaa Afirima, Nasir Umar

**Affiliations:** ^1^Bruyère Research Institute, Ottawa, Canada; ^2^London School of Hygiene and Tropical Medicine, London, UK; ^3^Social Work and Social Welfare, Staffordshire University, Stoke-on-Trent, Staffordshire, UK; ^4^Department of Obstetrics and Gynecology, University of Calabar, Calabar, Nigeria; ^5^APIN Public Health Initiative, Abuja, Nigeria; ^6^Pathfinder International, Abuja, Nigeria; ^7^FHI360, Lilongwe, Malawi; ^8^Catholic Relief Services, Abuja, Nigeria

## Abstract

**Background:**

Poor maternal health indices, including high maternal mortality, are among Nigeria's major public health problems. Most of these deaths can be prevented by timely access and utilization of maternity healthcare services by women. *Aim/Objective*. This study seeks to identify factors affecting the utilization of health facilities for the delivery of babies among mothers in Calabar, Cross River State, Nigeria. *Methodology*. The study was a community-based cross-sectional study. A structured questionnaire was administered to 422 women of reproductive age residents in the study area who had given birth at least once within the last five years prior to the survey using a multistage random sampling technique. Data generated were entered, coded, and analyzed using Statistical Packages for Social Sciences (SPSS version 22.0), and results were presented in tables and charts. Chi-squared tests and multiple logistic regression were used for the identification of variables associated with health facility-based delivery.

**Result:**

The mean age of respondents was 27.3 years (SD = 8.4). Fifty-two percent of the respondents utilized the health facility for delivery, 89.6% attended at least one antenatal clinic (ANC), and 18.9% completed at least 3 ANC sessions. There was a statistically significant association between health facility delivery and marital status (*P*=0.007), education (*P*=0.042), and family size (*P*=0.002). Older women (OR = 0.7, CI = 0.169–3.714), Christians (OR = 1.9, CI = 0.093–41.1), divorcees (OR = 3.7, CI = 0.00–0.00), and respondents who registered early (first trimester) for ANC (OR = 4.9, CI = 0.78–31.48) were found to be higher users of delivery services at the health facility.

**Conclusion:**

Community health intervention focusing on improving the knowledge and awareness of the significance of utilizing available delivery services at the healthcare facility should be developed and implemented.

## 1. Introduction

Complications during childbirth over the years have been largely responsible for most maternal deaths in the African region [1]. This is often exacerbated in poor-resource settings where access to maternal healthcare services is poor. Pregnancy and childbirth contribute to a low life expectancy of women in this region and constitute a significant threat to neonatal and infant survival [2].

The World Health Organization (WHO) estimated that about 810 women die every day in 2017 from obstetric complications, and developing countries contribute 94% of the total maternal death in the world [3]. In Nigeria, the Maternal Mortality Ratio (MMR) is currently 512 maternal deaths per 100,000 live births and is ranked the fourth highest globally [4, 5].

Complications arising during labor, delivery, and the postpartum period largely account for most maternal mortality [6]. The WHO noted that 75% of maternal mortality is primarily caused by obstetric hemorrhage, hypertension in pregnancy, infection, obstructed labor, and unsafe abortion [7]. Available evidence has shown that health facility-based delivery of babies by skilled birth attendants can reduce maternal mortality [6].

Most deliveries still occur at home without the assistance of a skilled birth attendant in developing countries [8]. Thus, although there has been a noticeable decline in maternal deaths by 38% from 342,000 in 2000 to 211,000 in 2017, the annual reduction rate of 2.9% is less than the required target of 6.4% to achieve the Sustainable Development Goal of 70 maternal deaths per 100,000 live births [9].

One in every 37 women faces a lifetime risk of dying during pregnancy or childbirth in Sub-Saharan Africa [7]. It has also been documented that 80% of global maternal deaths occur in Sub-Saharan Africa and Southern Asia [7]. However, delivery by skilled birth attendants was reportedly low, with 40% in Sub-Saharan Africa compared to 96% in developed countries [10]. Similarly, in Nigeria, delivery by skilled birth attendants is 43.3% (9% by doctors and 32% by midwives/nurses) [4].

In low- and middle-income countries, the prevalence of health facility-based delivery of infants ranged from 23% in the Republic of Chad to 94% in the Gabon republic [11]. Despite the free maternal health service policy in Kenya and Ghana, 47.6% of deliveries happen in a health facility in Kenya and 59% in Ghana, respectively [12]. In Eritrea, 16% of women residing in rural communities patronize health facilities for delivery compared to 73.2% in urban areas [13].

In Nigeria, national and subregional variation exists in the prevalence of health facility-based deliveries. However, from the national outlook, 36% of deliveries occur in the health facility, and in the South South region of Nigeria, 50.1% of deliveries occur in the health facility [14]. In this light, the current study seeks to identify factors affecting the use of health facilities for childbirth among mothers in Calabar metropolis, Cross River State, Nigeria.

## 2. Methodology

### 2.1. Research Design

This was a community-based cross-sectional study. The study utilized a structured questionnaire adapted from Nigeria Demographic Health Survey [4] and a cross-sectional survey conducted in Ethiopia [15]. The questionnaire was pretested prior to the study in a community similar to and outside the study area.

### 2.2. Study Setting

The study was carried out in Calabar, the capital of Cross River State, Nigeria. It has an airport and a seaport. It has a population of 371,022 (National Population Census, 2006). There are two Local Government Areas (LGAs) in Calabar, namely, Calabar Municipal and Calabar South. The LGAs are well mapped into 10 and 12 wards (the smallest administrative unit within the LGA).

There are two tertiary hospitals (the University of Calabar Teaching Hospital and the Federal Neuropsychiatric Hospital), two General hospitals, many primary health centers, registered clinics, registered maternity homes, and numerous private hospitals involved in maternal-newborn care. The major occupations of the people are trading, farming, fishing, and civil service.

### 2.3. Study Population

The study population comprised women of reproductive age (15–49 years) who delivered a live baby at least once in the past five years prior to the study and are permanent residents in the study area (Calabar metropolis).

### 2.4. Sampling Technique

Considering the wide horizon of the study setting, a multistage sampling technique was used to select subjects for this study as follows:

  Phase I: a systematic random sampling technique was employed to select three wards from each of the two local government areas using the lottery method.
  Phase II: a simple random sampling technique was employed in each selected ward to select 8 settlements using the lottery method. Numbers were assigned to each settlement, folded, and put in envelopes. The first 8 settlements picked were recruited into the study (i.e., 6 wards × 8 settlements = 48 settlements).
  Phase III: in each selected settlement, simple random sampling techniques were used to select eligible households; that is, publicly available addresses were entered in excel random sample software, and 8 households per settlement were randomly selected (i.e., ∼8 households × 48 settlements = 384 persons).

### 2.5. Sample Size

The sample size was determined using the Kiss formula, which is expressed as follows: *n* = *Z*^2^*Pq*/*d*^2^, where *n* is sample size, Z is equal to 1.96 corresponding to 95% confidence interval (constant at 95% precision), *d* is the degree of accuracy set at 5%, *q* = 1−*p* (1–0.5 = 0.5) (proportion of women who had not delivered at the health facility), and *p* = 0.5 (proportion of women who had delivered at the health facility); hence, *n* = 1.96 × 1.96 × 0.5 × 0.5/0.05 × 0.05, *n* = 384.

To account for attrition and incompletely filled questionnaires, the computed sample size (384) was increased by 10%, which gave a final sample size of 422.

### 2.6. Inclusion Criteria

Women of reproductive age (15–49 years) who had delivered at least one baby in the past five years prior to the study and were permanent residents of the study area (Calabar) irrespective of the place and outcome of delivery were recruited into the study.

### 2.7. Exclusion Criteria

The exclusion criteria include women with a psychiatric problem or who are seriously ill at the time of data collection, women below the age of 15 years and above 49 years at the time of data collection, and women who did not consent to the study.

### 2.8. Data Collection Tools and Procedures

The household survey was then carried out using a structured questionnaire which constituted only closed-ended questions.

The questionnaires included sociodemographic, obstetric characteristics, delivery place, and practice of institutional delivery and were administered by an interviewer.

### 2.9. Ethical Consideration

Approval was obtained from the Cross River State Ministry of Health Research Ethics Committee. Participation in the study was voluntary without any form of coercion, and those who were not willing to partake in the study were excluded from being interviewed. Written informed consent was obtained from each participant.

### 2.10. Data Management

Data were entered and analyzed using Statistical Package for Social Sciences (SPSS) version 22.0. Results were expressed in percentages and presented in tables and charts. Chi-square was used to determine the association between variables at 0.05 level of significance, whereas logistic regression was used to determine independent predictors for health services for childbirth.

## 3. Results

### 3.1. Sociodemographic Characteristics of Respondents

The mean age was 27.3(SD = 8.4) years. Most of the respondents, 292 (69%), were 30 years and below. About half, 51.4%, of the respondents, had postsecondary school education. A vast majority (84.2%) were employed. Details of the sociodemographic characteristics are shown in Table 1.

### 3.2. Maternal Health Information

Findings on maternal health information showed that the majority of respondents (69.9%) received no education on maternal health, while 30.1% were oriented on maternal health. Respondents' sources of information on maternal health included healthcare workers (68%), traditional birth attendants (7.9%), friends/relatives (16.7%), and radio/television (7.1%).

Some of the respondents (39.1%) identified nutritional problems as the major health problems in the community. Findings from the survey revealed that a higher proportion of the respondents (96.7%) reported having a health facility in their communities. Similarly, the majority of the respondents (90.9%) affirmed that the distance of the nearest health facility from their homes was less than one hour.

### 3.3. Obstetric Characteristics

**Figure 1:**
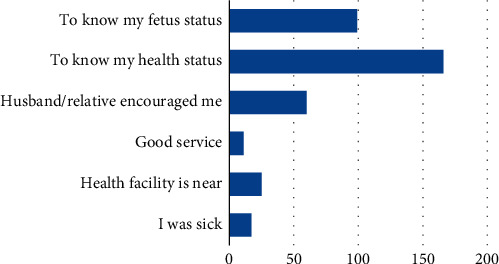
Reasons for attending ANC sessions.

**Figure 2:**
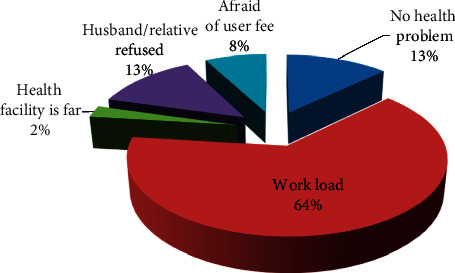
Reasons for not attending ANC session.

More than half (56.9%) of the respondents were multiparous (two or more children). The study revealed that 89.6% attended antenatal care (ANC), while 10.4% of respondents did not attend ANC. In enquiring for reasons for non-ANC attendance, 56.8% of the total respondents that did not participate in ANC attributed it to workload (Figures 1 and 2). Among the ANC attendees, 46% of them registered for ANC in the first trimester of pregnancy (0–13 weeks of gestation), 41% registered in the second trimester (14–26 weeks of gestation), while about 13% registered for ANC in the last trimester (27–40 weeks). Only 18.5% of respondents attended ANC three times and above. Maternal ill health was a major reason for early ANC registration.

Findings on sources of information on antenatal care for the recent pregnancy showed that a good proportion of the respondents (48.2%) got their information from government health centers, and a few of the respondents (1.8%) got their information from the traditional birth attendants. About 97% of the respondents received advice on where to deliver their babies during ANC visits. Also, a greater proportion of the respondents (90.7%) reported being told of the danger signs to watch out for during pregnancy.

### 3.4. Place of Delivery

The prevalence of health institutional delivery among the respondents was 52%. Of the remaining 48% who had childbirth outside health facilities, 72% of the respondents delivered in Traditional Birth Attendants' (TBAs) centers, while the rest delivered at their homes or worship centers. A greater proportion of the respondents (58.5%) decided by themselves where to give birth, while for about 20% of the respondents, the decision was made by their spouses or family members. However, a good proportion (87.4%) of the respondents agreed that giving birth in a health facility is better than giving birth at home because of cleanliness (5.9%) and shorter labor time (6.1%), in addition to saving baby's life (6.3%), saving mother's life (11%), and complication readiness in a health facility (70.7%). The majority of respondents (66.7%) attributed the reason for giving birth at home to “no need for transportation.” Other reasons for delivering at home included “no bleeding” (22.2%) and “no hospital cost” (11.1%) and about 0.9% of the respondents had difficulty with labor. The complications reported during labor were bleeding (50%), retained placenta (25%), and prolonged labor (25%).

About 53% of the respondents preferred to give birth at home in the subsequent pregnancy, while about 43% preferred to give birth in the health facility in the subsequent pregnancy. Also, a greater proportion of the respondents (82.2%) reported health facilities as their husbands preferred place of delivery. The majority of the respondents (69.7%) preferred to assist their mother/relatives during their next delivery. The most commonly used means of transportation to a health facility as reported by respondents included walking (45%), cars (41.5%), tricycles (6.4%), bus (4.9%), motorbikes (1.9%)

### 3.5. Predictors of Health Facility Delivery

The study revealed a statistically significant association between health facility delivery and marital status, education, and family size (see Table 2).

### 3.6. Logistic Regression Analysis

Multiple logistic regression analyses for odds of delivering in health facilities revealed that various factors play essential roles in determining the place for delivery. Older women are 0.7 times more likely to deliver in a health facility than young women (OR = 0.7, CI = 0.169–3.714). Divorcees are three times more likely to deliver in a health facility than single and married women (OR = 3.7, CI = 0.00–0.00). A partner's level of education can predict the choice of delivery in a health facility as respondents' partners with postsecondary education are 6 times more likely to deliver in a health facility than respondents with secondary education (OR = 6.4, CI = 0.57–72.47). Respondents who registered early (first trimester) for ANC are 4 times more likely to deliver in a health facility than those registered in the second and third trimester, respectively (OR = 4.9, CI = 0.78–31.48). Furthermore, the study revealed that respondents who received advice on where to deliver are 4 times more likely to deliver in a health facility (OR = 4.9, CI = 0.96–24.9) (Table 3).

## 4. Discussion

ANC attendance in many settings does not translate to effective utilization of health institutions for childbirth. Some women prefer to deliver at home; they visit the ANC during pregnancy and get registered to be accepted at the health facility should they be confronted with any possible obstetric problems during childbirth [16]. Some women attend ANC so that they may be able to access intervention programs such as HIV drugs, intermittent preventive treatment for malaria, and long-lasting insecticide nets, as well as immunization services such as administration of antitetanus toxoids [17].

In this study, more than three-quarters (89.6%) of the respondents reported having attended antenatal care at least once during their last pregnancy. This is higher than the Cross River State average of 72.6% [14]. Perhaps, this is because the study was conducted in an urban area of the state with a good number of the participants being educated and the availability and proximity of various categories of health institutions.

However, just over 18% of the ANC attendees completed at least three ANC sessions below the WHO recommendation of eight visits [1], and only 13 (3.4%) women attended at least four ANC sessions.

Access to antenatal care services by pregnant women is essential in determining the choice of place of delivery. Antenatal clinics provide an opportunity for well-trained skilled birth attendants to provide their clients with correct and factual information, especially as it concerns maternal and child health. During antenatal care, risk assessment is usually carried out and institutional delivery is recommended [18, 19].

As a global standard, pregnant women without any form of complications are encouraged to attend at least eight ANC visits spread across the three trimesters to access all the needed care and information concerning their health, especially as it concerns pregnancy, labor, and childbirth [1]. Early ANC registration and attendance are significantly accompanied by the benefits of early detection and prompt management of maternal problems and taking immediate corrective measures to benefit the pregnant woman and her unborn baby [1]. One key benefit of ANC is its positive association with health facility-based delivery and assistance by skilled birth attendants [18]. Women who utilized ANC services more than once were more likely to deliver at a health facility than mothers who did not attend ANC [19].

The prevalence of institutional delivery among the respondents was just 52%. This figure is similar to the 52.6% prevalence reported by Nigeria Demographic Health Survey [4]. There is a slight improvement from the findings of the national survey conducted in 2013, which noted an estimated 40 percent health facility delivery in the entire state [20].

### 4.1. Predictors of Health Facility Delivery

The study shows that sociodemographic factors such as maternal age, religion, occupation of partner, parity, and educational status were also major determinants of childbirth. This is in accordance with a population-based study conducted in Senegal which reported that the variations in the sociodemographic profile of women significantly influenced their choice of place of delivery [21].

Early ANC registration and regular visits may predict the probability of utilizing an orthodox health institution for childbirth. Access to other reproductive services during ANC may promote attendance and institutional service utilization [21, 22].

Furthermore, our study shows that multiparity is a negative predictor of hospital supervised delivery which is similar to a study in rural Tanzania which showed that utilization of health facility delivery was higher among women with low parity as compared to their high parity counterparts [23]. In contrast, the Ghana Demographic health survey analysis revealed that multiparous women had an increased tendency to deliver in a health facility than low parity women [24].

First trimester ANC registration largely accounts for the high rate of institutional delivery among the women compared to those who registered later. A systematic review in Ethiopia showed an increased likelihood of health facility delivery with early ANC registration [19]. This study also showed that women's employment status has a strong positive association with institutional delivery, where employed women were higher users of health facility delivery. This is in line with a cross-sectional study carried out in Sub-Saharan African countries [11]).

### 4.2. Limitations of the Study

This study did not explore women's views and perceptions of healthcare workers' attitudes towards pregnant women, including the quality of maternal care services they provide. Nevertheless, this could be one of the determining factors of health facility delivery.

The study was conducted in an urban area and cannot be generalized to rural settings.

## 5. Conclusion

In conclusion, the study revealed an association between social factors such as marital status, the mother's educational level, religion, and family size with the utilization of health facilities for delivery services. A key determining factor for better maternal and neonatal outcomes is the continual availability and regular use of reproductive health services provided by well-trained and certified healthcare providers.

## Figures and Tables

**Table 1:** Sociodemographic characteristics of respondents, *N* = 422.

Variable	Frequency	Percentage
*Religion*		
Christian	404	95.7
Muslim	16	3.8
Traditional African Religion	2	0.5
Total	422	100

*Age in years*		
15–20	118	28
21–25	92	22
26–30	82	19
31–35	57	13
36–40	34	8
41–49	39	10
Total	422	100

*Highest level of education*		
Postsecondary	217	51.4
Secondary school	186	44.1
Primary school	19	4.5
Total	422	100

*Employment status*		
Employed	198	47
Self-employed	157	37.2
Unemployed	67	15.8
Total	422	100

*Educational level of partner*		
Postsecondary	270	63.9
Secondary school	147	35
Primary school	5	1.1
Total	422	100

*Occupation of partner*		
Farmer	122	28.9
Daily laborer	54	12.8
Businessman	68	16.1
Civil servant	178	42.2
Total	422	100

**Table 2:** Predictors of health facility delivery.

Characteristics	Delivered in health facility	Delivered outside health facility	*χ* ^2^	Df	*p* value
*Religion*					
Christian	209	195	0.021	2	0.990
Muslim	8	8			
Traditional African Religion	1	1			
Total	218	204			

*Marital status*					
Single	120	141	12.068	3	0.007
Married	83	59			
Divorced/separated	8	3			
Cohabiting	7	1			
Total	218	204			

*Education*					
Postsecondary	102	115	6.326	2	0.042
Secondary school	102	84			
Primary school	14	5			
Total	218	204			

*Employment status*					
Employed	100	98	4.446	3	0.217
Self-employed	78	79			
Unemployed	42	25			
Total	218	204			

*Educational level of partner*					
Postsecondary	139	131	0.143	2	0.931
Secondary school	76	71			
Primary school	3	2			
Total	218	204			

*Occupation of partner*					
Farmer	55	67	11.539	3	0.009
Daily laborer	30	24			
Businessman	47	21			
Civil servant	86	92			
Total	218	204			

*Health facility accessibility*					
Health facility accessible	211	197	0.016	1	0.899
Health facility not accessible	7	7			
Total	218	204			

*Family size*					
One–three	141	127	11.668	4	0.020
Two–four	49	65			
Five–seven	24	8			
Eight–eleven	3	4			
More than eleven	1	0			
Total	218	204			

*ANC attendance*					
Attended ANC	194	184	0.164	1	0.686
Did not attend ANC	24	20			
Total	218	204			

*Number of pregnancies in the last five years*					
One	76	60	1.567	2	0.457
Two	121	119			
Three	21	24			
Total	218	203			

*Age of respondents*					
15–20 years	57	59	10.683	6	0.099
21–25 years	47	45			
26–30 years	34	49			
31–35 years	32	26			
36–40 years	21	13			
41–45 years	19	9			
46–49 years	8	3			
Total	218	204			

**Table 3:** Multiple logistic regression analysis to predict health facility delivery.

Predictors	Sig.	OR	95% confidence interval	
			Lower bound	Upper bound
*Age*				
15–20 years	0.148	0.362	0.092	1.434
21–25 years	0.186	0.392	0.098	1.570
26–30 years	0.059	0.260	0.064	1.052
31–35 years	0.287	0.462	0.111	1.918
36–40 years	0.511	0.606	0.136	2.705
41–45 years	0.767	0.792	0.169	3.714

*Religion*				
Christian	0.666	1.957	0.093	41.071
Muslim	0.668	0.469	0.015	14.992

*Marital status*				
Single	0.991	1.071	0.000	0.000
Married	0.991	1.976	0.000	0.000
Divorced/separated	0.992	3.709	0.000	0.000

*Educational status*				
Postsecondary	0.056	0.227	0.049	1.041
Secondary school	0.245	0.380	0.074	1.940

*Employment status*				
Employed	.000	7.972	4.047	1.570
Self-employed	.000	7.478	3.783	1.478
Unemployed	.	1.116	1.116	1.116

*Occupation of partner*				
Farmer	0.709	0.901	0.522	1.557
Daily laborer	0.560	1.275	0.563	2.886
Business man	0.104	1.854	0.880	3.904

*Partner's educational status*				
Postsecondary	0.130	6.463	0.576	72.475
Secondary school	0.217	4.979	0.390	63.516
Health facility available	0.722	0.629	0.049	8.089

*ANC registration*				
First trimester	0.089	4.966	0.783	31.483
Second trimester	0.764	1.313	0.223	7.734
Third trimester	0.670	1.478	0.245	8.914
Received advice where to deliver	0.056	4.902	0.961	24.998

## Data Availability

The numerical model simulations upon which this study is based are too large to archive or transfer. Instead, we provide all the information needed to replicate the simulations and can be found in the references. The model code, compilation script, initial and boundary condition files, and the namelist settings are available on the reference page.
